# Long non-coding RNA PCED1B-AS1 promotes pancreatic ductal adenocarcinoma progression by regulating the miR-411-3p/HIF-1α axis

**DOI:** 10.3892/or.2021.8085

**Published:** 2021-05-20

**Authors:** Yi Zhang, Huan Ma, Chang Chen

**Affiliations:** 1 Department of Gastroenterology, The People's Hospital of China Three Gorges University and The First People's Hospital of Yichang, Yichang, Hubei 443000, P.R. China; 2 Department of Gastroenterology, Qingdao Municipal Hospital, Qingdao, Shandong 266000, P.R. China

**Keywords:** pancreatic ductal adenocarcinoma, PC-esterase domain containing 1B-antisense RNA 1, microRNA-411-3p, hypoxia inducible factor-1α

## Abstract

An increasing number of studies have shown that long non-coding RNAs (lncRNAs) are crucially involved in tumorigenesis. However, the biological functions, underlying mechanisms and clinical value of lncRNA PC-esterase domain containing 1B-antisense RNA 1 (PCED1B-AS1) in pancreatic ductal adenocarcinoma (PDAC) have not been determined, to the best of our knowledge. In the present study, the expression of PCED1B-AS1, microRNA (miR)-411-3p and hypoxia inducible factor (HIF)-1α mRNA in 47 cases of PDAC tissues were detected using reverse transcription-quantitative (RT-q)PCR. Moreover, the effects of PCED1B-AS1 on the biological behaviors of PDAC cells were assessed using Cell Counting Kit-8, EdU staining and Transwell assays. Bioinformatics analysis, RT-qPCR, western blotting, dual luciferase reporter gene and RNA immunoprecipitation assays were performed to determine the regulatory relationships between PCED1B-AS1, miR-411-3p and HIF-1α. We demonstrated that PCED1B-AS1 was significantly upregulated in PDAC tumor tissues, and its expression was associated with advanced Tumor-Node-Metastasis stage and lymph node metastasis. PCED1B-AS1 knockdown inhibited PDAC cell proliferation, invasion as well as epithelial-mesenchymal transition (EMT) *in vitro*. Mechanistically, PCED1B-AS1 was shown to target miR-411-3p, resulting in the upregulation of HIF-1α. In conclusion, PCED1B-AS1 expression was upregulated in PDAC tissues and cells, and it participated in promoting the proliferation, invasion and EMT of cancer cells by modulating the miR-411-3p/HIF-1α axis.

## Introduction

Pancreatic cancer has a 5-year survival rate less than 8% and is one of the deadliest types of cancer worldwide. Pancreatic ductal adenocarcinoma (PDAC) is the most common pathological type of pancreatic cancer (1–4). For patients with advanced stage PDAC, therapeutic options are limited, and their prognosis is extremely poor (1–4). Thus, there is an urgent need to improve the current understanding of the mechanisms underlying progression of PDAC to identify novel therapeutic targets.

Long non-coding RNAs (lncRNAs) are a type of RNA molecule of >200 nucleotides in length, which have limited or no protein-coding capabilities (5). Previously, it was hypothesized that lncRNAs were transcriptional noise, and that they did not possess any biological function (5). However, in the last decade, a growing number of studies have demonstrated that lncRNAs participate in a range of cellular biological processes, including cell proliferation, migration, differentiation and apoptosis (6–8). lncRNAs also exhibit crucial roles in the development and/or progression of cancers. For example, knockdown of lncRNA actin filament associated protein 1-antisense RNA 1 was found to impede the proliferation and cell cycle progression of colon cancer cells (9). It has been reported that lncRNA TMPO antisense RNA 1 (TMPO-AS1) expression is upregulated in bladder cancer tissues and cells, where it promotes cell growth, migration and invasion (10). In non-small lung cancer, knockdown of lncRNA colon cancer associated transcript 1 suppressed cancer cell proliferation and sensitized cancer cells to gefitinib (11). lncRNA PC-esterase domain containing 1B antisense RNA 1 (PCED1B-AS1) was found to be involved in the regulation of macrophage apoptosis and autophagy in active tuberculosis (12,13); additionally, it has been demonstrated that PCED1B-AS1 is abnormally expressed in gliomas and breast cancer tissues, where it functions as an oncogenic lncRNA (14,15). However, its biological function, mechanistic partners and clinical value in PDAC have not been assessed.

lncRNAs can function as competitive endogenous RNAs (ceRNAs), competitively interacting with microRNAs (miRNAs/miRs) and indirectly regulating the expression of target genes (16,17). For example, lncRNA Pvt1 oncogene acts as a molecular sponge, absorbing miR-448 and upregulating SERPINE1 mRNA binding protein 1, thus promoting pancreatic cancer cell proliferation and migration (18). It has also been reported that lncRNA X inactive specific transcript can facilitate the migration, invasion and epithelial-mesenchymal transition (EMT) of pancreatic cancer cells by repressing miR-429, indirectly resulting in upregulation of zinc finger E-box binding homeobox 1 expression (19). Moreover, highly upregulated in liver cancer (HULC) was found to promote the proliferation, migration and invasion of pancreatic cancer cells by downregulating miR-15a and activating the PI3K/AKT pathway (20). These studies suggest that lncRNAs act as ceRNAs and participate in the progression of PDAC.

Hypoxia-inducible factor-1α (HIF-1α), a dominant regulator of a tumor cell's response to hypoxia (21), is closely associated with the progression and metastasis of several types of cancer, including PDAC (22–24). In the present study, it was shown that PCED1B-AS1 expression was significantly upregulated in PDAC tissues and cell lines. PCED1B-AS1 overexpression facilitated the malignant biological behaviors of cancer cells. Mechanistically, it acted as a ceRNA of miR-411-3p, resulting in upregulation of HIF-1α. The results of the present study clarify the mechanism by which HIF-1α expression is dysregulated in PDAC, and identified PCED1B-AS1 as a novel oncogenic lncRNA in PDAC.

## Materials and methods

### 

#### Tissue sample collection

A total of 47 pairs of PDAC tissue samples and the corresponding adjacent normal tissues were surgically removed from patients between January 2017 and January 2019 from The People's Hospital of Three Gorges University and collected. The patients had a mean age of 45 years (range, 28–77 years; 22 male and 25 female) and did not receive any radiotherapy or chemotherapy prior to surgery. Written informed consent was provided by each patient and the collection of human samples was approved by the Ethics Committee of the People's Hospital of Three Gorges University. All tissues were stored in liquid nitrogen (−196°C).

#### Cell culture and transfection

Five PDAC cell lines (AsPC-1, PANC-1, CFPAC-1, SW1990 and BxPC-3), normal human pancreatic ductal epithelial cell line HPDE6-C7, and human embryonic kidney cell line, 293T, were all purchased from The Cell Bank Type Culture Collection of the Chinese Academy of Sciences. Cells were cultured in DMEM (Gibco; Thermo Fisher Scientific, Inc.) supplemented with 10% FBS (Invitrogen; Thermo Fisher Scientific, Inc.) in a humidified incubator at 37°C with 5% CO_2_.

Small interfering (si)RNAs targeting PCED1B-AS1 (si-PCED1B-AS1), negative control siRNAs (si-NC), miR-411-3p mimic, mimic negative control (mimic NC), miR-411-3p inhibitor, inhibitor negative control (NC), HIF-1α overexpression plasmid, and the negative control plasmid were purchased from Shanghai GenePharma Co., Ltd.. According to the manufacturer's protocols, PDAC cells were transfected using Lipofectamine^®^ 2000 transfection reagent (Invitrogen; Thermo Fisher Scientific, Inc.). Cells were collected for subsequent analysis 48 h after the transfection.

#### Reverse transcription-quantitative (RT-q) PCR

TRIzol^®^ (Invitrogen; Thermo Fisher Scientific, Inc.) was used to obtain total RNA from PDAC tissues and cells. Total RNA was reverse transcribed into cDNA using a PrimeScript RT kit (Takara Bio, Inc.). qPCR was performed using a SYBR-Green PCR MasterMix kit (Takara Bio, Inc.) on an ABI 7500 real-time PCR system (Applied Biosystems; Thermo Fisher Scientific, Inc.). Expression of PCED1B-AS1 and HIF-1α was normalized to GAPDH. Expression of miR-411-3p was normalized to U6. The relative expression level of each gene was quantified using the 2^−ΔΔCq^ method (25). The sequences of the primers are listed in Table I.

#### Dual luciferase reporter assay

A dual luciferase reporter assay was performed using the 293T cell line. First, the target sites of miR-411-3p on PCED1B-AS1 or HIF-1α 3′ untranslated region (3′UTR) were predicted using bioinformatics analysis. The wild-type (WT) and mutant (MUT) PCED1B-AS1 and HIF-1α 3′UTR regions were amplified and inserted into a pmir-GLO luciferase reporter vector (Promega Corp.). The recombinant plasmids PCED1B-AS1-WT, PCED1B-AS1-MUT, HIF-1α-WT and HIF-1α-MUT were subsequently co-transfected into 293T cells with the miR-411-3p mimic or NC mimic, respectively. After 48 h, the luciferase activities were measured using a Dual-Luciferase Reporter Assay system (Promega Corp.) according to the manufacturer's protocol.

#### RNA-binding protein immunoprecipitation (RIP) assay

PDAC cells transfected with miR-411-3p mimics or NC mimics were collected, and according to the manufacturer's protocols, RIP was performed using an anti-Ago2 antibody (EMD Millipore) and an RIP assay kit (EMD Millipore). Mouse anti-human immunoglobulin G (IgG) antibody was used as the control. Subsequently, RNA was extracted using TRIzol, and the expression of PCED1B-AS1 was assessed using RT-qPCR.

#### Cell Counting Kit-8 (CCK-8) assay

The viability of PDAC cells was detected using a CCK-8 assay (Beyotime Institute of Biotechnology). CFPAC-1 and SW1990 cells were plated into a 96-well plate. After 24, 48, 72 and 96 h, 10 µl of CCK-8 solution was added to each well, and the cells were further incubated at 37°C for 2 h. Subsequently, the absorbance of each well was assessed at an optical density of 450 nm using a microplate reader. A proliferation curve was plotted with time as the abscissa and the value of absorbance as the ordinate.

#### EdU staining assay

Cell proliferation was also evaluated using an EdU assay. Transfected cells were plated in a 96-well plate (5×10^3^ cells/well) and cultured for 24 h. Then, 100 µl of EdU solution (50 µM; Guangzhou RiboBio Co., Ltd.) was added to each well, and the cells were subsequently incubated at 37°C for 2 h. Cells were washed 3 times with PBS and then fixed using paraformaldehyde/glycine for 30 min. Cells were permeabilized using 0.5% Triton X-100, then stained with Apollo fluorescent staining reaction solution for 30 min in the dark and washed twice with methanol and PBS. Cells were subsequently counterstained with DAPI staining solution for 30 min, and washed with PBS 3 times. Fluorescence was observed using a fluorescence microscope, and the percentage of EdU-positive cells was counted and calculated. Cell proliferation rate=number of EdU-positive cells/number of DAPI-positive cells ×100%.

#### Transwell assay

Transwell chambers (8-µm pore size; BD Biosciences) were used to assess the invasive ability of PDAC cells. CFPAC-1 and SW1990 cells were suspended in serum-free DMEM and added to the upper chamber, which had been pre-coated with Matrigel. The lower chamber was filled with 600 µl of medium supplemented with 10% FBS. A total of 24 h after incubation at 37°C, the chambers were removed, and the residual cells remaining on the upper surface of the membrane were wiped off using a cotton swab. The cells which had invaded to the lower surface of the membrane were fixed using 4% paraformaldehyde, and stained using 0.1% crystal violet for 10 min. Membranes were washed using tap water and dried, and the cells were observed using an inverted microscope and counted. The number of cells from five fields in each well were counted, and the experiments were performed in triplicate.

#### Western blotting

PDAC cells were lysed using RIPA lysis buffer (Beyotime Institute of Biotechnology). An equivalent amount of protein was loaded per lane on SDS-gel (stacking gel 4%, separation gel 10%), resolved using SDS-PAGE, transferred to PVDF membranes (EMD Millipore) and blocked using 5% skimmed milk. Subsequently, the membranes were incubated with the primary antibodies overnight at 4°C. The primary antibodies used were: Anti-HIF-1α antibody (cat. no. ab51608; 1:1,000; Abcam), anti-N-cadherin antibody (cat. no. ab202030; 1:1,000; Abcam), anti-E-cadherin antibody (cat. no. ab40772; 1:1,000; Abcam), anti-Vimentin antibody (cat. no. ab92547; 1:1,000; Abcam), anti-Snail antibody (cat. no. ab53519; 1:1,000; Abcam) or anti-β-actin antibody (cat. no. ab179467; 1:2,000; Abcam). Subsequently, the membranes were incubated with an HRP-conjugated secondary antibody (cat. no. ab205718; 1:2,000; Abcam) at room temperature for 1 h. Signals were visualized using an ECL kit (Beyotime Institute of Biotechnology). Densitometry analysis was performed using ImageJ_v1.8.0 (National Institutes of Health).

#### Bioinformatics analysis

The expression pattern of PCED1B-AS1 in PAAD and normal tissues was predicted using the Gene Expression Profiling Interactive Analysis (GEPIA) database (http://gepia.cancer-pku.cn/) (26). The potential target miRNAs of PCED1B-AS1 was predicted using the LncBase Predicted version 2 database (http://carolina.imis.athena-innovation.gr/diana_tools/web/index.php?r=lncbasev2%2Findex) (27). The interaction between HIF-1α and miR-411-3p was predicted using the TargetScan database (http://www.targetscan.org/vert_72/) (28).

#### Statistical analysis

Statistical analysis was performed using SPSS version 21.0 (IBM Corp.). Data are presented as the mean ± standard deviation of at least three independent experiments. Distribution of the data was examined using a Kolmogorov-Smirnov test. A two-tailed student's t-test was used to determine the differences between two groups. A one-way ANOVA with Tukey's post-hoc test was used to determine the differences among ≥3 groups. For data that were not normally distributed, comparison of expression in PDAC tissue samples and the corresponding adjacent normal tissues was performed using a paired sample Wilcoxon signed-rank test. A χ^2^ test was used to analyze the association between the expression of PCED1B-AS1 and the clinicopathological characteristics of patients with PDAC. Pearson's correlation coefficient analysis was utilized to determine the correlation between PCED1B-AS1 expression and miR-411-3p expression or HIF-1α expression.

## Results

### 

#### PCED1B-AS1 expression is upregulated in pancreatic cancer tissues and cells

**Figure 1.**
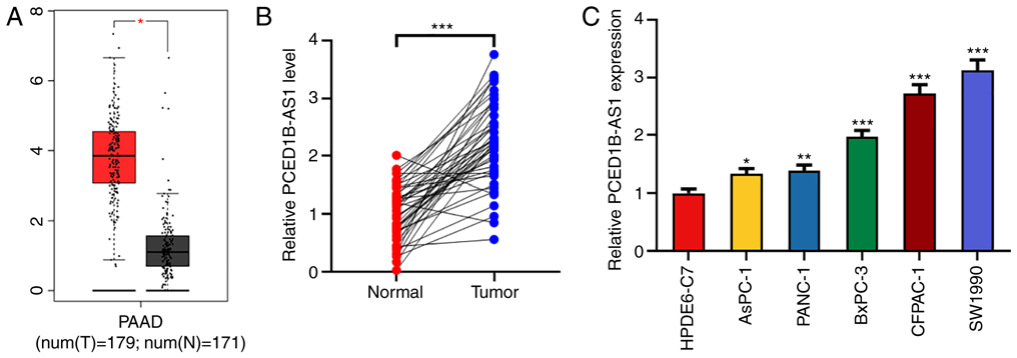
PCED1B-AS1 is upregulated in PDAC tissues and cells. (A) Expression levels of PCED1B-AS1 in the PDAC tissues based on data obtained from GEPIA. (B) Relative expression levels of PCED1B-AS1 in 47 cases of PDAC tissues and the corresponding non-tumor tissues were detected using RT-qPCR. (C) RT-qPCR was used to detect the expression levels of PCED1B-AS1 in the normal human pancreatic ductal epithelial cell line HPDE6-C7 and the five PDAC cell lines, AsPC-1, PANC-1, CFPAC-1, SW1990 and BxPC-3. *P<0.05, **P<0.01, ***P<0.001 vs. normal tissues or the HPDE6-C7 cell line. GEPIA, Gene Expression Profiling Interactive Analysis; PDAC, pancreatic ductal adenocarcinoma; PCED1B-AS1, PC-esterase domain containing 1B-antisense RNA 1; RT-qPCR, reverse transcription-quantitative PCR.

Using the Gene Expression Profiling Interactive Analysis (GEPIA) database, 171 cases of normal tissues and 179 cases of cancerous tissues were compared. PCED1B-AS1 expression was significantly higher in the 179 PDAC tissues (Fig. 1A). To confirm the upregulation of PCED1B-AS1 in PDAC tissues, the expression of PCED1B-AS1 in PDAC tissues and corresponding non-tumor tissues was further examined using RT-qPCR. Compared with the corresponding non-tumor tissues, the expression of PCED1B-AS1 was upregulated in PDAC tissues (Fig. 1B). To assess the association between the expression of PCED1B-AS1 and the clinicopathological characteristics of 47 patients with PDAC, patients were divided into a high expression group (n=25) and low expression group (n=22), based on the median expression level of PCED1B-AS1. The results demonstrated that increased expression of PCED1B-AS1 was positively correlated with advanced TNM stage (stage III–IV) and lymph node metastasis (Table II). Additionally, the expression levels of PCED1B-AS1 in PDAC cell lines (AsPC-1, PANC-1, CFPAC-1, SW1990 and BxPC-3) was significantly higher compared with the normal pancreatic ductal epithelial cell line HPDE6-C7 (Fig. 1C). Among the five PDAC cell lines, PCED1B-AS1 expression was highest in CFPAC-1 and SW1990 cells, thus these two cell lines were used for subsequent experiments.

#### PCED1B-AS1 knockdown reduces proliferation, invasion and EMT of pancreatic cancer cells

**Figure 2.**
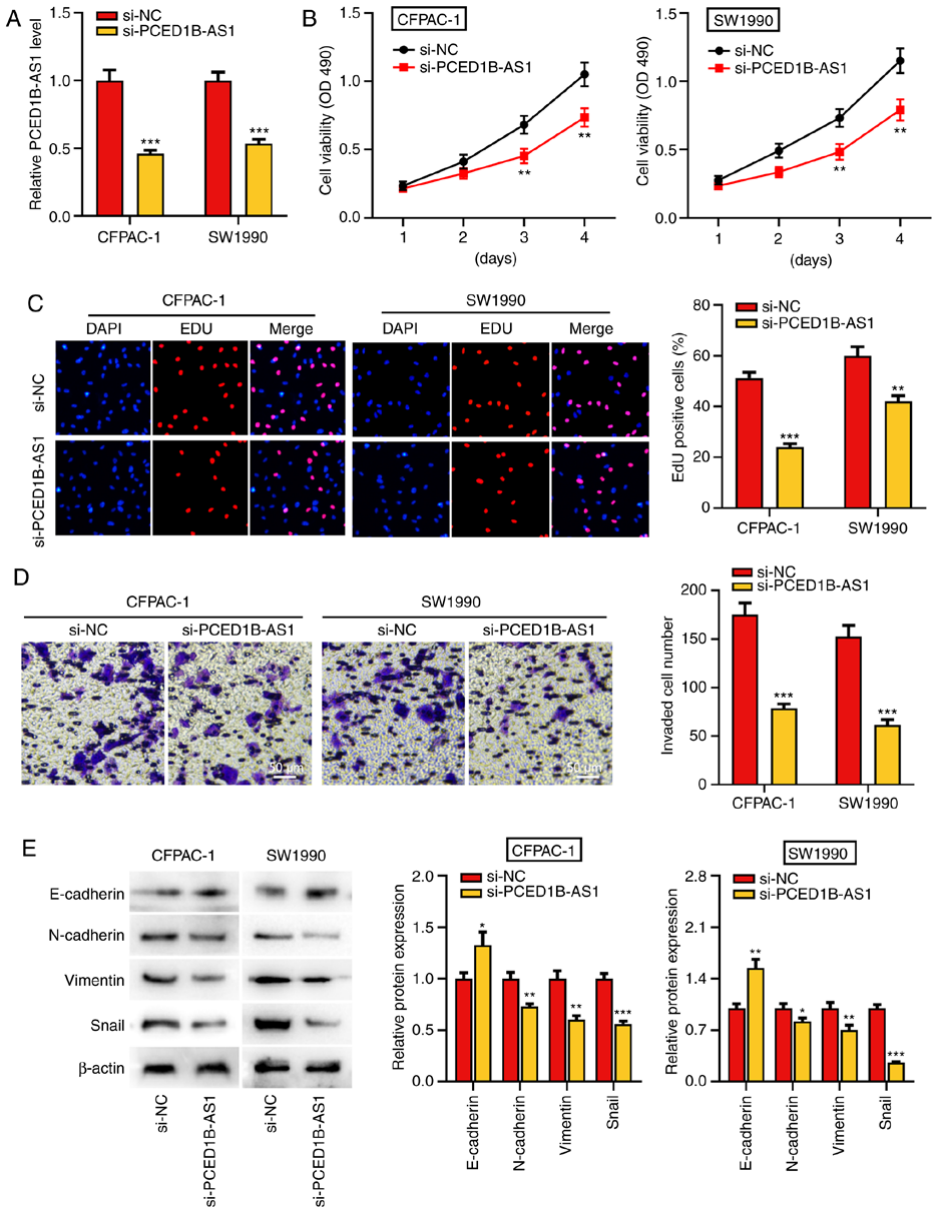
PCED1B-AS1 knockdown represses PDAC cell proliferation, invasion, and EMT. (A) RT-qPCR was utilized to investigate the expression of PCED1B-AS1 in CFPAC-1 and SW1990 cells transfected with si-NC or si-PCED1B-AS1. (B and C) CCK-8 and EdU staining assay were used to assess the effect of PCED1B-AS1 knockdown on proliferation of CFPAC-1 and SW1990 cells. (D) Transwell invasion assays were used to assess the effects of PCED1B-AS1 knockdown on the invasion of CFPAC-1 and SW1990 cells. (E) Western blotting was used to assess the expression of the EMT markers, E-cadherin, N-cadherin, Vimentin and Snail, following transfection. *P<0.05, **P<0.01, ***P<0.001 vs. si-NC. siRNA, small interfering RNA; si-NC, si-negative control; PDAC, pancreatic ductal adenocarcinoma; PCED1B-AS1, PC-esterase domain containing 1B-antisense RNA 1; RT-qPCR, reverse transcription-quantitative PCR; EMT, epithelial-mesenchymal transition; CCK-8, Cell Counting Kit-8.

To further study the biological function of PCED1B-AS1 on the progression of PDAC, si-PCED1B-AS1 was transfected into CFPAC-1 and SW1990 cells to knockdown its expression. Compared with the si-NC group, transfection of si-PCED1B-AS1 significantly reduced the expression of PCED1B-AS1 in CFPAC-1 and SW1990 cells, suggesting that PCED1B-AS1 was successfully knocked down in CFPAC-1 and SW1990 cells (Fig. 2A). The results of the CCK-8 and EdU assay revealed that the PCED1B-AS1 knockdown significantly reduced PDAC cell proliferation (Fig. 2B and C). Transwell invasion assays and western blotting were used to determine the effect of PCED1B-AS1 knockdown on cell invasion and EMT. Compared with cells transfected with si-NC, CFPAC-1 and SW1990 cells transfected with si-PCED1B-AS1 exhibited significantly reduced invasion (Fig. 2D). Furthermore, knockdown of PCED1B-AS1 significantly increased the expression of E-cadherin, and significantly reduced the expression of N-cadherin, Vimentin and Snail in both PDAC cell lines (Fig. 2E).

#### PCED1B-AS1 negatively regulates miR-411-3p expression

**Figure 3.**
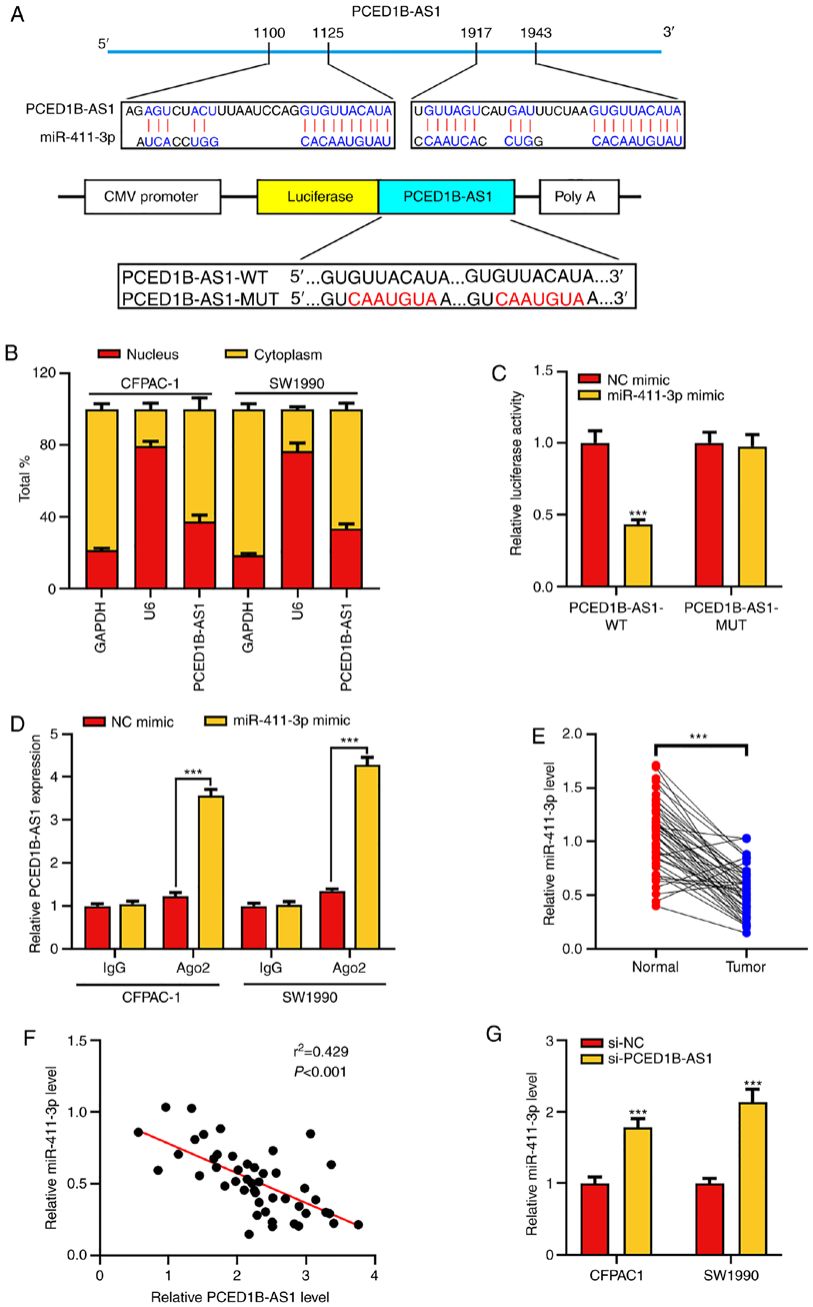
PCED1B-AS1 sponges miR-411-3p. (A) Predicted binding sites between miR-411-3p and PCED1B-AS1, and the WT and MUT sequences. (B) Expression of PCED1B-AS1 in the nuclei and cytoplasm of CFPAC-1 and SW1990 cells was evaluated using RT-qPCR. (C) 293T cells were co-transfected with miR-411-3p or NC mimic and luciferase reporter vectors containing PCED1B-AS1 WT or MUT. The relative luciferase activity of cells was measured. (D) Direct binding between miR-411-3p and PCED1B-AS1 in CFPAC-1 and SW1990 cells was examined using RIP experiments. (E) Relative expression levels of miR-411-3p in the 47 PDAC tissues and the corresponding non-tumor tissues were detected using RT-qPCR. (F) Correlation analysis of miR-411-3p and PCED1B-AS1 expression in the 47 PDAC patients was analyzed using Pearson's correlation analysis. (G) RT-qPCR was used to investigate the expression of miR-411-3p in CFPAC-1 and SW1990 cells transfected with si-NC or si-PCED1B-AS1. ***P<0.001. siRNA, small interfering RNA; si-NC, si-negative control; PDAC, pancreatic ductal adenocarcinoma; PCED1B-AS1, PC-esterase domain containing 1B-antisense RNA 1; RT-qPCR, reverse transcription-quantitative PCR; WT, wild-type; MUT, mutant; RIP, RNA immunoprecipitation; miR, microRNA.

To elucidate whether PCED1B-AS1 acts as a ceRNA in the progression of PDAC, the online bioinformatics tool LncBase Predicted version 2 was used to predict the potential target miRNAs of PCED1B-AS1 (Table SI). The results indicated that miR-411-3p was a potential target of PCED1B-AS1 (Fig. 3A). The subcellular localization of PCED1B-AS1 in CFPAC-1 and SW1990 cells was then determined. RT-qPCR results showed that PCED1B-AS1 was primarily expressed in the cytoplasm of CFPAC-1 and SW1990 cells (Fig. 3B). Subsequently, dual luciferase reporter assays and RIP experiments were used to confirm the targeted binding. The results showed that miR-411-3p mimics could reduce the luciferase activity of cells in the PCED1B-AS1-WT group, but had no significant effect on the PCED1B-AS1-MUT group (Fig. 3C). RIP analysis further confirmed increased enrichment of miR-411-3p and PCED1B-AS1 in the Ago2-immunoprecipitation complex (Figs. 3D and S1). Additionally, compared with the non-tumor tissues, miR-411-3p was significantly downregulated in PDAC tissues (Fig. 3E). The expression levels of PCED1B-AS1 in PDAC tissues was negatively correlated with the expression levels of miR-411-3p (Fig. 3F). Compared with the si-NC transfected control group, PCED1B-AS1 knockdown significantly increased the expression of miR-411-3p in PDAC cell lines (Fig. 3G). These results suggest that PCED1B-AS1 can effectively reduce the expression of miR-411-3p.

#### HIF-1α is a target gene of miR-411-3p

**Figure 4.**
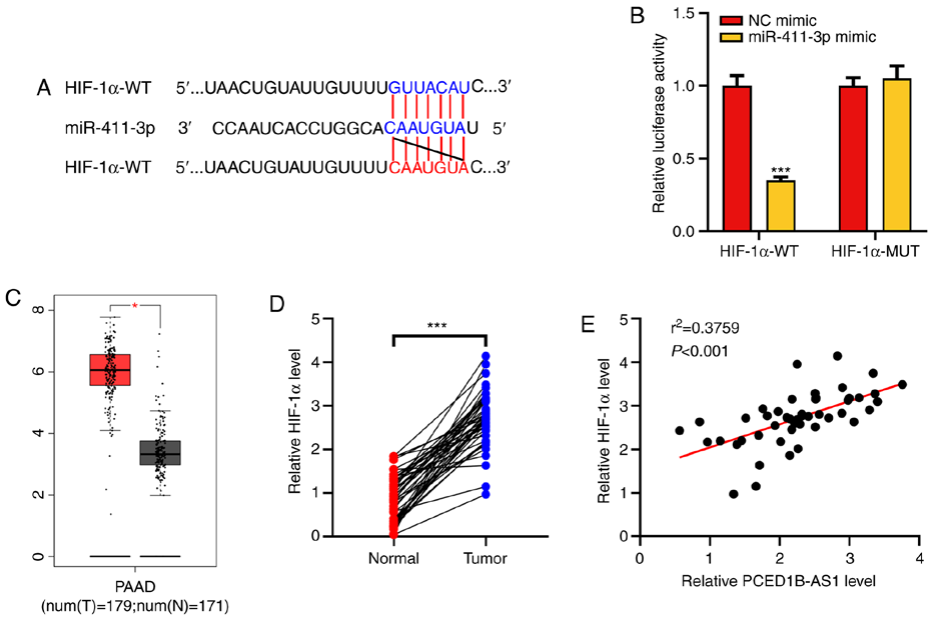
PCED1B-AS1 functions as a ceRNA of miR-411-3p to regulate HIF-1α expression. (A) Binding sequence between the HIF-1α 3′UTR and miR-411-3p was predicted using bioinformatics. (B) Dual luciferase reporter assays showed that miR-411-3p targeted the HIF-1α 3′-UTR. (C) Analysis of the expression of HIF-1α in PDAC tissues in the GEPIA database. (D) Relative expression levels of miR-411-3p in the 47 PDAC tissues and corresponding non-tumor tissues were detected using RT-qPCR. (E) Correlation between the HIF-1α expression and PCED1B-AS1 expression in clinical samples was analyzed using Pearson's correlation analysis. ***P<0.001. ceRNA, competing endogenous RNA; miR, microRNA; HIF-1α, hypoxia inducible factor-1α; UTR, untranslated region; PDAC, pancreatic ductal adenocarcinoma; GEPIA, Gene Expression Profiling Interactive Analysis; PCED1B-AS1, PC-esterase domain containing 1B-antisense RNA 1.

HIF-1α was predicted as a potential target for miR-411-3p using the TargetScan database (Fig. 4A). Dual-luciferase reporter assays were then performed to confirm this prediction. It was demonstrated that following co-transfection with the miR-411-3p mimics, the luciferase activity of HIF-1α-WT reporter was significantly reduced, whereas the luciferase activity of HIF-1α-MUT reporter was not altered (Fig. 4B). Analysis of data obtained from the GEPIA database showed that HIF-1α expression was upregulated in PDAC tissues (Fig. 4C). The expression of HIF-1α mRNA in the clinical PDAC tissues was then determined using RT-qPCR. Its expression was significantly higher in tumor tissues of patients with PDAC and was positively correlated with the expression of PCED1B-AS1 (Fig. 4D and E). These results suggest that miR-411-3p can target HIF-1α expression, and PCED1B-AS1 may exert its biological functions via a miR-411-3p/HIF-1α axis.

#### PCED1B-AS1 regulates the miR-411-3p/HIF-1α axis to reduce PDAC cell proliferation, invasion and EMT

**Figure 5.**
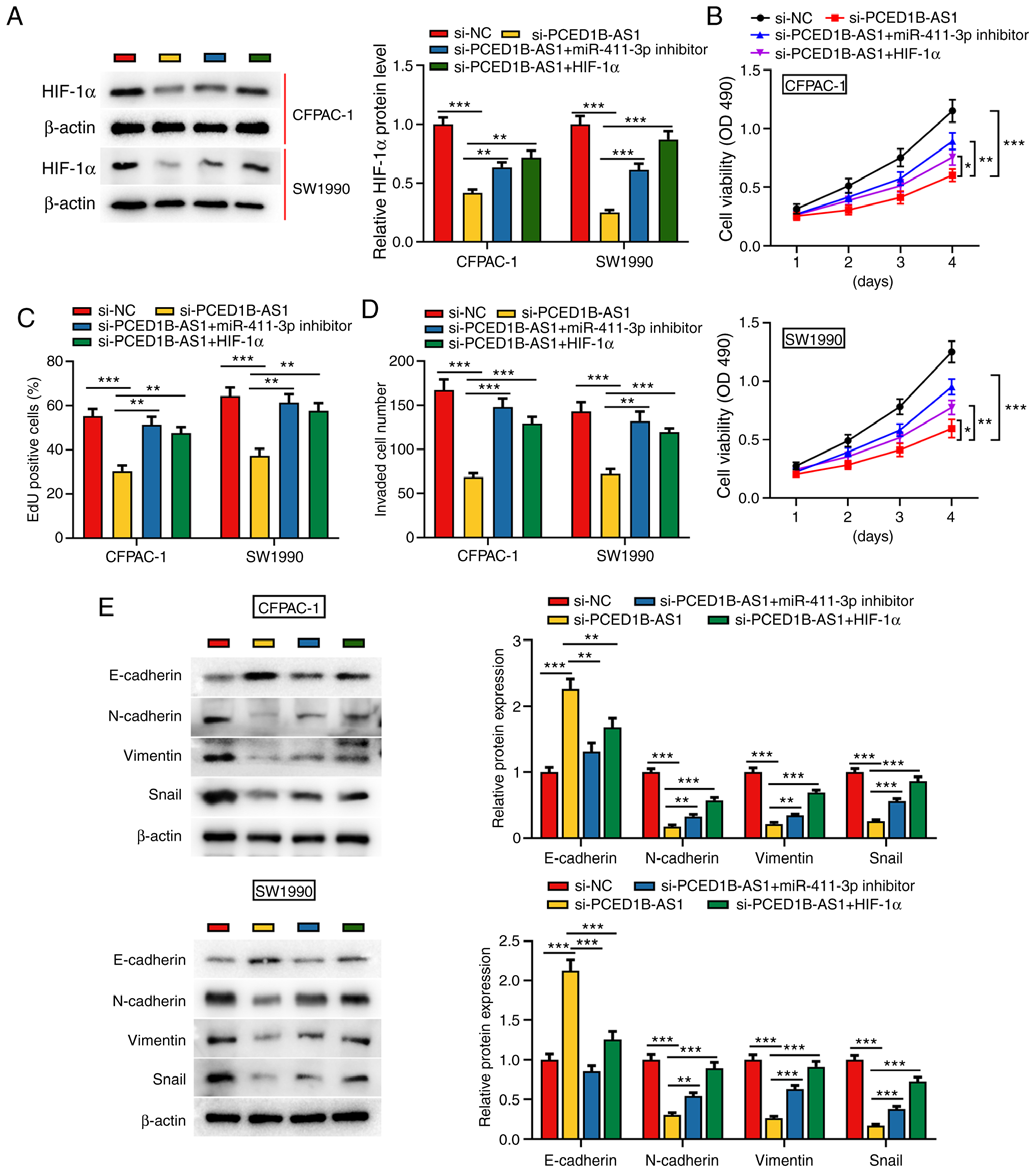
PCED1B-AS1 modulates the biological behaviors of PDAC cells via regulation of a miR-411-3p/HIF-1α axis. (A) Western blotting was used to detect the expression of HIF-1α expression in CFPAC-1 and SW1990 cells transfected with si-NC, si-PCED1B-AS1, si-PCED1B-AS1 + miR-411-3p inhibitor or si-PCED1B-AS1 + HIF-1α overexpression plasmid. (B-E) Proliferation, invasion and expression of EMT markers in CFPAC-1 and SW1990 cells were detected using a CCK-8 assay, EdU assay, Transwell invasion assay and western blotting, respectively. *P<0.05, **P<0.01, ***P<0.001. siRNA, small interfering RNA; si-NC, si-negative control; PDAC, pancreatic ductal adenocarcinoma; PCED1B-AS1, PC-esterase domain containing 1B-antisense RNA 1; miR, microRNA; HIF-1α, hypoxia inducible factor-1α; CCK-8, Cell Counting Kit-8.

To further investigate the effect of PCED1B-AS1 and miR-411-3p on the biological behaviors of PDAC cells, si-PCED1B-AS1, miR-411-3p inhibitor or HIF-1α overexpression plasmids were co-transfected into CFPAC-1 and SW1990 cells (Figs. S2 and S3). Western blotting was used to investigate the expression of HIF-1α; PCED1B-AS1 knockdown significantly reduced the expression of HIF-1α in CFPAC-1 and SW1990 cells, whereas the transfection of miR-411-3p inhibitor and HIF-1α overexpression plasmid restored the expression of HIF-1α (Fig. 5A). Cell proliferation, invasion and EMT in each group were then evaluated. CCK-8 and EdU staining assays demonstrated that compared with the control group, the proliferation of CFPAC-1 and SW1990 cells in the si-PCED1B-AS1 group was reduced; however, this reduction was reversed by the transfection of miR-411-3p inhibitor or HIF-1α overexpression plasmid (Fig. 5B and C). Transwell assays and western blotting were used to assess invasion and EMT. In the si-PCED1B-AS1 transfected cells, invasion and EMT were reduced, and co-transfection with the miR-411-3p inhibitor or HIF-1α attenuated this inhibitory effect (Fig. 5D and E). These results show that PCED1B-AS1 modulates PDAC cell proliferation, invasion and EMT via regulation of a miR-411-3p/HIF-1α axis.

## Discussion

Pancreatic cancer is one of the most aggressive and fatal types of cancer (1–4). An increasing number of studies have shown that non-coding RNAs, such as long non-coding RNAs (lncRNAs) and microRNAs (miRNAs), serve prominent roles in regulating the occurrence and progression of pancreatic ductal adenocarcinoma (PDAC). The present study showed that lncRNA PC-esterase domain containing 1B-antisense RNA 1 (PCED1B-AS1) expression is upregulated in PDAC tissues and cell lines, and it is closely associated with TNM stage and lymph node metastasis of the patients. Additionally, PCED1B-AS1 knockdown impaired proliferation, invasion and EMT of PDAC cells. These results show that PCED1B-AS1 functions as an oncogenic lncRNA in PDAC.

lncRNAs can function as competitive endogenous RNA (ceRNAs), regulating the expression and function of miRNAs (29). For example, as a ceRNA for miR-520a-3p, lncRNA non-coding RNA activated by DNA damage (NORAD) was found to modulate the PI3K/AKT/mTOR signaling pathway to promote the occurrence and progression of non-small cell lung cancer (30). It has been reported that lncRNA ADPGK-AS1 upregulates orthodenticle homeobox 1 expression, promotes breast cancer cell proliferation and migration, induces EMT, and impedes apoptosis by sponging miR-3196 (31). PCED1B-AS1 is upregulated in several types of cancer (14,15). In gliomas, PCED1B-AS1 promotes cancer cell proliferation and reduces apoptosis by modulating a miR-194-5p/PCED1B axis (14). In the present study, bioinformatics analysis, luciferase reporter gene experiments and RIP experiments confirmed that PCED1B-AS1 directly interacted with miR-411-3p in PDAC. Furthermore, PCED1B-AS1 knockdown reduced PDAC cell proliferation, invasion and EMT; conversely, co-transfection with miR-411-3p inhibitors reversed these effects. These data suggest that PCED1B-AS1 regulates PDAC proliferation, invasion and EMT by sponging miR-411-3p.

MiR-411-3p is a tumor-suppressive miRNA. Low expression of miR-411-3p is significantly correlated with reduced overall survival in patients with non-small cell lung cancer (32). It has also been reported that CDKN2B-AS1 interacts with miR-411-3p and regulates ovarian cancer progression via a HIF-1α/VEGF/p38 pathway (33). In the present study, it was confirmed through bioinformatics analysis and luciferase reporter gene assays that HIF-1α was a direct target of miR-411-3p in PDAC, and that hypoxia inducible factor-1α (HIF-1α) was positively regulated by PCED1B-AS1. Previous studies report that HIF-1α is involved in regulating the malignant biological behaviors of cancer cells, such as cell proliferation, migration and angiogenesis, in several types of cancer (34–38). For example, HIF-1α is upregulated in colorectal cancer cell lines and contributes to angiogenesis by modulating the expression of EMT-related molecules claudin-4, E-cadherin and Vimentin (38). In pancreatic cancer, HIF-1α expression has been reported to be upregulated, and it is involved in the regulation of the Warburg effect, cancer metastasis and chemoresistance; upregulated expression of HIF-1α is associated with unfavorable prognosis of the patients (39–41). A recent study showed that ascorbate inhibits tumor growth of PDAC by reducing the expression of HIF-1α at the protein level under hypoxic condition via post-translational regulation (42). HIF-1α regulates granulocyte-macrophage colony-stimulating factor (GM-CSF) expression via direct binding to the hypoxia response element in the promoter region of GM-CSF gene, and participates in tumor-nerve interaction in PDAC (43). In addition, HIF-1α can directly bind to the hypoxia response element in the promoter region of cyclophilin A, regulating cyclophilin A expression and thus promoting PDAC cell proliferation and invasion, and suppressing apoptosis *in vitro* (44). In the present study, it was found that HIF-1α was significantly upregulated in PDAC tissues, consistent with previous reports (39–41). The upregulation of HIF-1α was primarily due to the presence of a hypoxic tumor microenvironment (21). Importantly, in the present study, it was also demonstrated that the expression of HIF-1α was regulated by a PCED1B-AS1/miR-411-3p axis. Functional experiments showed that HIF-1α overexpression partially reversed the inhibition of PCED1B-AS1 knockdown on PDAC cell proliferation, invasion and EMT. These results may explain the mechanism by which HIF-1α expression is dysregulated in PDAC.

In summary, it was demonstrated that PCED1B-AS1 was significantly upregulated in PDAC tissues and PDAC cell lines, and it was associated with a less favorable outcome in patients with PDAC. PCED1B-AS1 knockdown impeded PDAC cell proliferation, invasion and EMT. PCED1B-AS1 was shown to directly target miR-411-3p, acting as a ceRNA, indirectly increasing HIF-1α expression, thereby promoting PDAC progression. Collectively, these results provide an improved understanding of the characteristics of the PCED1B-AS1/miR-411-3p/HIF-1α axis in PDAC progression, which may provide novel directions for improvement of PDAC diagnosis and treatment. In future studies, *in vivo* models will be used to further validate the findings of the present study. Additionally, a larger cohort will be enrolled and survival analysis will be performed to evaluate the potential of PCED1B-AS1 as a biomarker in PDAC.

## Supplementary Material

Supporting Data

Supporting Data

## Figures and Tables

**Table I.** Sequences of the primers used for RT-qPCR.

Gene	Sequence, 5′-3′
PCED1B-AS1	
Forward	TTTGATGTTGGCCAATGCCG
Reverse	GGGCAGGGAGTCTTCATAGC
HIF-1α	
Forward	AGTAATCGGACTACCGGACGTG
Reverse	TGGGCATTACATCGCATGCATC
GAPDH	
Forward	GTCAGGATCCACTCATCACG
Reverse	GATCGGACTTACGGACTCACATC
microRNA-411-3p	
Forward	TAGTAGACCGTATAGCGTACG
U6	
Forward	AAAGACCTGTACGCCAACAC
Reverse	GTCATACTCCTGCTTGCTGAT

PCED1B-AS1, PC-esterase domain containing 1B-antisense RNA 1; HIF-1α, hypoxia inducible factor-1α.

**Table II.** Correlation between PCED1B-AS1 expression levels and the clinicopathologic characteristics of the 47 patients with PDAC.

		PCED1B-AS1 expression		
			
Clinicopathological characteristic	n	High, n=25	Low, n=22	P-value
Age, years				0.282
≥60	21	13	8	
<60	26	12	14	
Sex				0.106
Male	22	15	8	
Female	25	10	14	
Tumor size, cm				0.119
>2	27	17	10	
≤2	20	8	12	
Differentiation				0.191
Poor	24	15	9	
Moderate and well	23	10	13	
Tumor-Node-Metastasis stage				0.028^a^
I+II	14	4	10	
III+IV	33	21	12	
Distant metastasis				0.118
Negative	33	20	13	
Positive	14	5	9	
Lymph node metastasis				0.0141^a^
Absent	21	7	14	
Present	26	18	8	

a P<0.05. PDAC, pancreatic ductal adenocarcinoma; PCED1B-AS1, PC-esterase domain containing 1B-antisense RNA 1.

## Data Availability

The datasets used during the present study are available from the corresponding author upon reasonable request.

## References

[1] Siegel RL, Miller KD, Jemal A (2017). Cancer statistics, 2017. CA Cancer J Clin.

[2] Steeg PS (2016). Targeting metastasis. Nat Rev Cancer.

[3] Heestand GM, Kurzrock R (2015). Molecular landscape of pancreatic cancer: Implications for current clinical trials. Oncotarget.

[4] Melisi D, Calvetti L, Frizziero M, Tortora G (2014). Pancreatic cancer: Systemic combination therapies for a heterogeneous disease. Curr Pharm Des.

[5] Ponting CP, Oliver PL, Reik W (2009). Evolution and functions of long noncoding RNAs. Cell.

[6] Wu H, Yang L, Chen LL (2017). The diversity of long noncoding RNAs and Their Generation. Trends Genet.

[7] Zhou X, Liu S, Cai G, Kong L, Zhang T, Ren Y, Wu Y, Mei M, Zhang L, Wang X (2015). Long non coding RNA MALAT1 promotes tumor growth and metastasis by inducing epithelial-mesenchymal transition in oral squamous cell carcinoma. Sci Rep.

[8] Khaitan D, Dinger ME, Mazar J, Crawford J, Smith MA, Mattick JS, Perera RJ (2011). The melanoma-upregulated long noncoding RNA SPRY4-IT1 modulates apoptosis and invasion. Cancer Res.

[9] Tang J, Zhong G, Wu J, Chen H, Jia Y (2018). Long noncoding RNA AFAP1-AS1 facilitates tumor growth through enhancer of zeste homolog 2 in colorectal cancer. Am J Cancer Res.

[10] Luo H, Yang L, Liu C, Wang X, Dong Q, Liu L, Wei Q (2020). TMPO-AS1/miR-98-5p/EBF1 feedback loop contributes to the progression of bladder cancer. Int J Biochem Cell Biol.

[11] Jin X, Liu X, Zhang Z, Guan Y (2020). lncRNA CCAT1 Acts as a MicroRNA-218 sponge to increase gefitinib resistance in NSCLC by targeting HOXA1. Mol Ther Nucleic Acids.

[12] Li M, Cui J, Niu W, Huang J, Feng T, Sun B, Yao H (2019). Long non-coding PCED1B-AS1 regulates macrophage apoptosis and autophagy by sponging miR-155 in active tuberculosis. Biochem Biophys Res Commun.

[13] Zhao Y, Wang Z, Zhang W, Zhang L (2019). MicroRNAs play an essential role in autophagy regulation in various disease phenotypes. Biofactors.

[14] Yang J, Yu D, Liu X, Changyong E, Yu S (2020). LncRNA PCED1B-AS1 activates the proliferation and restricts the apoptosis of glioma through cooperating with miR-194-5p/PCED1B axis. J Cell Biochem.

[15] Yuan CL, Jiang XM, Yi Y, E JF, Zhang ND, Luo X, Zou N, Wei W, Liu YY (2019). Identification of differentially expressed lncRNAs and mRNAs in luminal-B breast cancer by RNA-sequencing. BMC Cancer.

[16] Ye Y, Li SL, Wang SY (2018). Construction and analysis of mRNA, miRNA, lncRNA, and TF regulatory networks reveal the key genes associated with prostate cancer. PLoS One.

[17] Duguang L, Jin H, Xiaowei Q, Peng X, Xiaodong W, Zhennan L, Jianjun Q, Jie Y (2017). The involvement of lncRNAs in the development and progression of pancreatic cancer. Cancer Biol Ther.

[18] Zhao L, Kong H, Sun H, Chen Z, Chen B, Zhou M (2018). LncRNA-PVT1 promotes pancreatic cancer cells proliferation and migration through acting as a molecular sponge to regulate miR-448. J Cell Physiol.

[19] Shen J, Hong L, Yu D, Cao T, Zhou Z, He S (2019). LncRNA XIST promotes pancreatic cancer migration, invasion and EMT by sponging miR-429 to modulate ZEB1 expression. Int J Biochem Cell Biol.

[20] Feng H, Wei B, Zhang Y (2019). Long non-coding RNA HULC promotes proliferation, migration and invasion of pancreatic cancer cells by down-regulating microRNA-15a. Int J Biol Macromol.

[21] Puppo M, Battaglia F, Ottaviano C, Delfino S, Ribatti D, Varesio L, Bosco MC (2008). Topotecan inhibits vascular endothelial growth factor production and angiogenic activity induced by hypoxia in human neuroblastoma by targeting hypoxia-inducible factor-1alpha and −2alpha. Mol Cancer Ther.

[22] Ren W, Mi D, Yang K, Cao N, Tian J, Li Z, Ma B (2013). The expression of hypoxia-inducible factor-1α and its clinical significance in lung cancer: A systematic review and meta-analysis. Swiss Med Wkly.

[23] Hung JJ, Yang MH, Hsu HS, Hsu WH, Liu JS, Wu KJ (2009). Prognostic significance of hypoxia-inducible factor-1alpha, TWIST1 and Snail expression in resectable non-small cell lung cancer. Thorax.

[24] Wu YL, Hu LN, Zheng CD, Sun RC, Zhang SX, Yan Q, Li YX (2016). Expression of hypoxia-inducible factor 1α in gastric cancer and its clinical signficance. Zhonghua Yi Xue Za Zhi.

[25] Livak KJ, Schmittgen TD (2001). Analysis of relative gene expression data using real-time quantitative PCR and the 2(-Delta Delta C(T)) method. Methods.

[26] Tang Z, Li C, Kang B, Gao G, Li C, Zhang Z (2017). GEPIA: A web server for cancer and normal gene expression profiling and interactive analyses. Nucleic Acids Res.

[27] Paraskevopoulou MD, Vlachos IS, Karagkouni D, Georgakilas G, Kanellos I, Vergoulis T, Zagganas K, Tsanakas P, Floros E, Dalamagas T, Hatzigeorgiou AG (2016). DIANA-LncBase v2: Indexing microRNA targets on non-coding transcripts. Nucleic Acids Res.

[28] Agarwal V, Bell GW, Nam JW, Bartel DP (2015). Predicting effective microRNA target sites in mammalian mRNAs. Elife.

[29] Abdollahzadeh R, Daraei A, Mansoori Y, Sepahvand M, Amoli MM, Tavakkoly-Bazzaz J (2019). Competing endogenous RNA (ceRNA) cross talk and language in ceRNA regulatory networks: A new look at hallmarks of breast cancer. J Cell Physiol.

[30] Wan Y, Yao Z, Chen W, Li D (2020). The lncRNA NORAD/miR-520a-3p facilitates malignancy in non-small cell lung cancer via PI3k/Akt/mTOR signaling pathway. Onco Targets Ther.

[31] Yang J, Wu W, Wu M, Ding J (2019). Long noncoding RNA ADPGK-AS1 promotes cell proliferation, migration, and EMT process through regulating miR-3196/OTX1 axis in breast cancer. In VitroCell Dev Biol Anim.

[32] Halvorsen AR, Sandhu V, Sprauten M, Flote VG, Kure EH, Brustugun OT, Helland Å (2018). Circulating microRNAs associated with prolonged overall survival in lung cancer patients treated with nivolumab. Acta Oncol.

[33] Wang Y, Huang Y, Liu H, Su D, Luo F, Zhou F (2019). Long noncoding RNA CDKN2B-AS1 interacts with miR-411-3p to regulate ovarian cancer in vitro and in vivo through HIF-1a/VEGF/P38 pathway. Biochem Biophys Res Commun.

[34] Lu Y, Li Y, Wang Z, Xie S, Wang Q, Lei X, Ruan Y, Li J (2019). Downregulation of RGMA by HIF-1A/miR-210-3p axis promotes cell proliferation in oral squamous cell carcinoma. Biomed Pharmacother.

[35] Sohn SH, Kim B, Sul HJ, Choi BY, Kim HS, Zang DY (2020). Foretinib inhibits cancer stemness and gastric cancer cell proliferation by decreasing CD44 and c-MET signaling. Onco Targets Ther.

[36] Ma D, Fan SB, Hua N, Li GH, Chang Q, Liu X (2020). Hypermethylation of single CpG dinucleotides at the promoter of CXCL13 gene promoting cell migration in cervical cancer. Curr Cancer Drug Targets.

[37] Yang QC, Zeng BF, Shi ZM, Dong Y, Jiang ZM, Huang J, Lv YM, Yang CX, Liu YW (2006). Inhibition of hypoxia-induced angiogenesis by trichostatin A via suppression of HIF-1a activity in human osteosarcoma. J Exp Clin Cancer Res.

[38] Li W, Zong S, Shi Q, Li H, Xu J, Hou F (2016). Hypoxia-induced vasculogenic mimicry formation in human colorectal cancer cells: Involvement of HIF-1a, claudin-4, and E-cadherin and Vimentin. Sci Rep.

[39] Shen Y, Chen G, Zhuang L, Xu L, Lin J, Liu L (2019). ARHGAP4 mediates the Warburg effect in pancreatic cancer through the mTOR and HIF-1α signaling pathways. Onco Targets Ther.

[40] Shukla SK, Purohit V, Mehla K, Gunda V, Chaika NV, Vernucci E, King RJ, Abrego J, Goode GD, Dasgupta A (2017). MUC1 and HIF-1alpha signaling crosstalk induces anabolic glucose metabolism to impart gemcitabine resistance to pancreatic cancer. Cancer Cell.

[41] Colbert LE, Fisher SB, Balci S, Saka B, Chen Z, Kim S, El-Rayes BF, Adsay NV, Maithel SK, Landry JC, Curran WJ (2015). High nuclear hypoxia-inducible factor 1 alpha expression is a predictor of distant recurrence in patients with resected pancreatic adenocarcinoma. Int J Radiat Oncol Biol Phys.

[42] Wilkes JG, O'Leary BR, Du J, Klinger AR, Sibenaller ZA, Doskey CM, Gibson-Corley KN, Alexander MS, Tsai S, Buettner GR, Cullen JJ (2018). Pharmacologic ascorbate (P-AscH-) suppresses hypoxia-inducible Factor-1α (HIF-1α) in pancreatic adenocarcinoma. Clin Exp Metastasis.

[43] Wang H, Jia R, Zhao T, Li X, Lang M, Lan C, Wang H, Li Z, Zhou B, Wu L (2019). HIF-1α mediates tumor-nerve interactions through the up-regulation of GM-CSF in pancreatic ductal adenocarcinoma. Cancer Lett.

[44] Zhang H, Chen J, Liu F, Gao C, Wang X, Zhao T, Liu J, Gao S, Zhao X, Ren H, Hao J (2014). CypA, a gene downstream of HIF-1α, promotes the development of PDAC. PLoS One.

