# Supplementary material for: Long non-coding RNA PCED1B-AS1 promotes pancreatic ductal adenocarcinoma progression by regulating the miR-411-3p/HIF-1α axis
Source: Oncol Rep. 2021 May 20;46(1):134. doi: 10.3892/or.2021.8085 (PMC8144929; doi:10.3892/or.2021.8085)

Figure S1. RT-qPCR was utilized to investigate the expression of miR-411-3p in CFPAC-1 and SW1990 cells transfected with NC mimic or miR-411-3p mimic. ***P<0.001 vs. NC mimic.

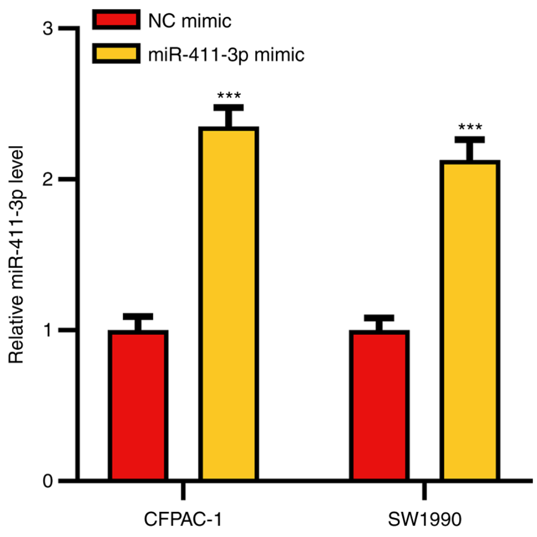

Figure S2. RT-qPCR was utilized to investigate the expression of miR-411-3p in CFPAC-1 and SW1990 cells transfected with NC inhibitor or miR-411-3p inhibitor. ***P<0.001 vs. NC inhibitor.

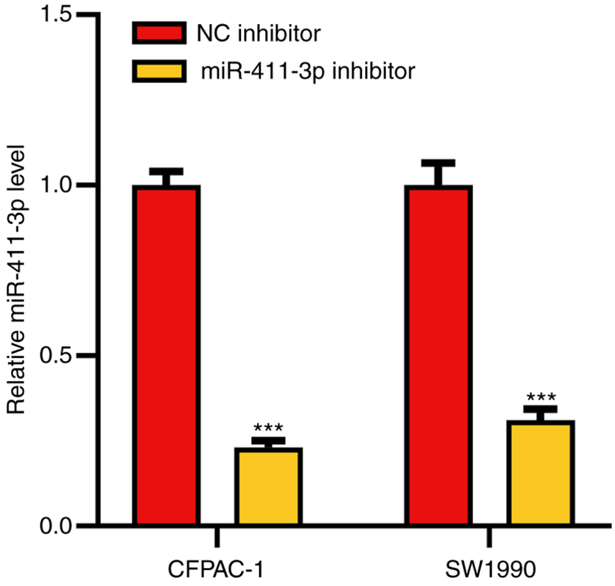

Figure S3. RT-qPCR was utilized to investigate the expression of HIF-α in CFPAC-1 and SW1990 cells transfected with NC or HIF-1α overexpression plasmid. $^{***}$P<0.001 vs. NC. HIF-1α, hypoxia inducible factor-1α.

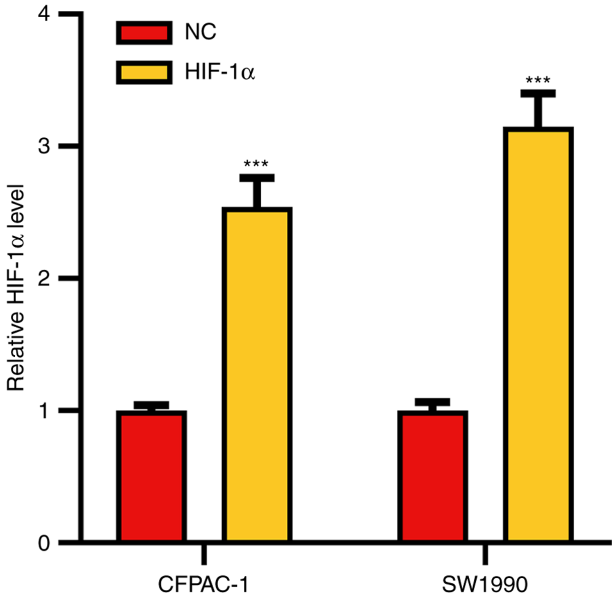

Supplement: Supporting Data [file Supplementary_Data1.pdf]
